# Is there a difference in plate positioning for clavicle shaft fractures? A retrospective cohort study of 355 cases

**DOI:** 10.1007/s00068-026-03140-5

**Published:** 2026-03-12

**Authors:** Michelle Antonia Hinkelmann, Désirée Schibler, Flurina Frei, Florin Allemann, Hans-Christoph Pape, Christiane Barthel

**Affiliations:** 1 Department of Trauma Surgery, University Hospital of Zurich, Zürich, Switzerland; 2 University of Zurich, Zürich, Switzerland; 3 Department of Shoulder and Elbow Surgery, Schulthess Klinik, Lengghalde 2, Zürich, CH-8008 Switzerland

**Keywords:** Clavicle shaft fracture, Open reduction and internal fixation, Plate osteosynthesis, Callus formation, Implant failure

## Abstract

**Purpose:**

Plate osteosynthesis is a common procedure for clavicle shaft fractures. The plate can be placed in an anterior, anterior-superior, or superior position. This study aims to evaluate surgical treatment options regarding different plate positions for clavicle shaft fractures and to compare them regarding patient and perioperative characteristics, frequency of use, appearance of callus, complication rates and implant removal.

**Methods:**

A retrospective cohort study of patients treated with plate osteosynthesis after clavicle shaft fracture between January 2016 and December 2024 at a Swiss level 1 trauma center was performed. Patients aged ≥ 16 years treated with anterior, anterior-superior or superior plate placement and availability of clinical follow-up data with X-ray were included. Presence of callus was subdivided into early (≤ 6 weeks), intermediate (> 6 to ≤ 26 weeks), and late (> 26 weeks).

**Results:**

A total of 355 cases with a median of 41 years (range 17 to 90 years) (81.7%, *n* = 290 males and 18.3%, *n* = 65 females) were analyzed. The most commonly used plate position was anterior (92.4%, *n* = 328), followed by superior (5.6%, *n* = 20) and anterior-superior (2%, *n* = 7). Plate placement had no significant influence on operation time, callus formation, implant removal, or refracture after implant removal. Complications occurred in 6.2% (*n* = 22) of cases with a trend towards an increased complication rate with anterior-superior plate position (28.6%, *n* = 2), but the incidence of complications did not differ when comparing only anterior (5.5%, *n* = 18) and superior plate placement (10%, *n* = 2).

**Conclusion:**

All three plate positions are valid surgical treatments for clavicle shaft fractures as similar results were observed with anterior, anterior-superior and superior plate placement. Further studies, particularly on anterior-superior plate fixation as well as patient reported outcome measures and clinical follow-up, are necessary to achieve optimal long-term surgical results.

**Level of evidence:**

Level III, retrospective comparative study.

## Background

Clavicle fractures are among the most common fractures in adults. Most fractures occur in the middle third of the clavicle (76–81%), followed by lateral third fractures (17–21%) and medial third fractures (2–3%) [1, 2]. The subdivision corresponds to the commonly used Allman classification [3].

Surgery is generally recommended for displaced midshaft fractures because it achieves significantly higher union rates (operative ~ 96.7% versus conservative ~ 88.9%; number needed to treat ~ 10 to prevent one nonunion) and markedly reduces nonunion risk (relative risk ≈ 0.17) [4, 5]. The socioeconomic impact is substantial, as operative treatment significantly shortens time to union (operative 12.7–18 weeks versus conservative 20.0–30 weeks, *p* < 0.001) and accelerates return to work [4, 6]. Common surgical approaches include plate fixation and, in certain cases, intramedullary fixation without significant differences in clinical outcome and complication rates [7–10]. The optimal configuration of plate osteosynthesis - particularly with respect to plate position - remains insufficiently defined.

**Fig. 1:**
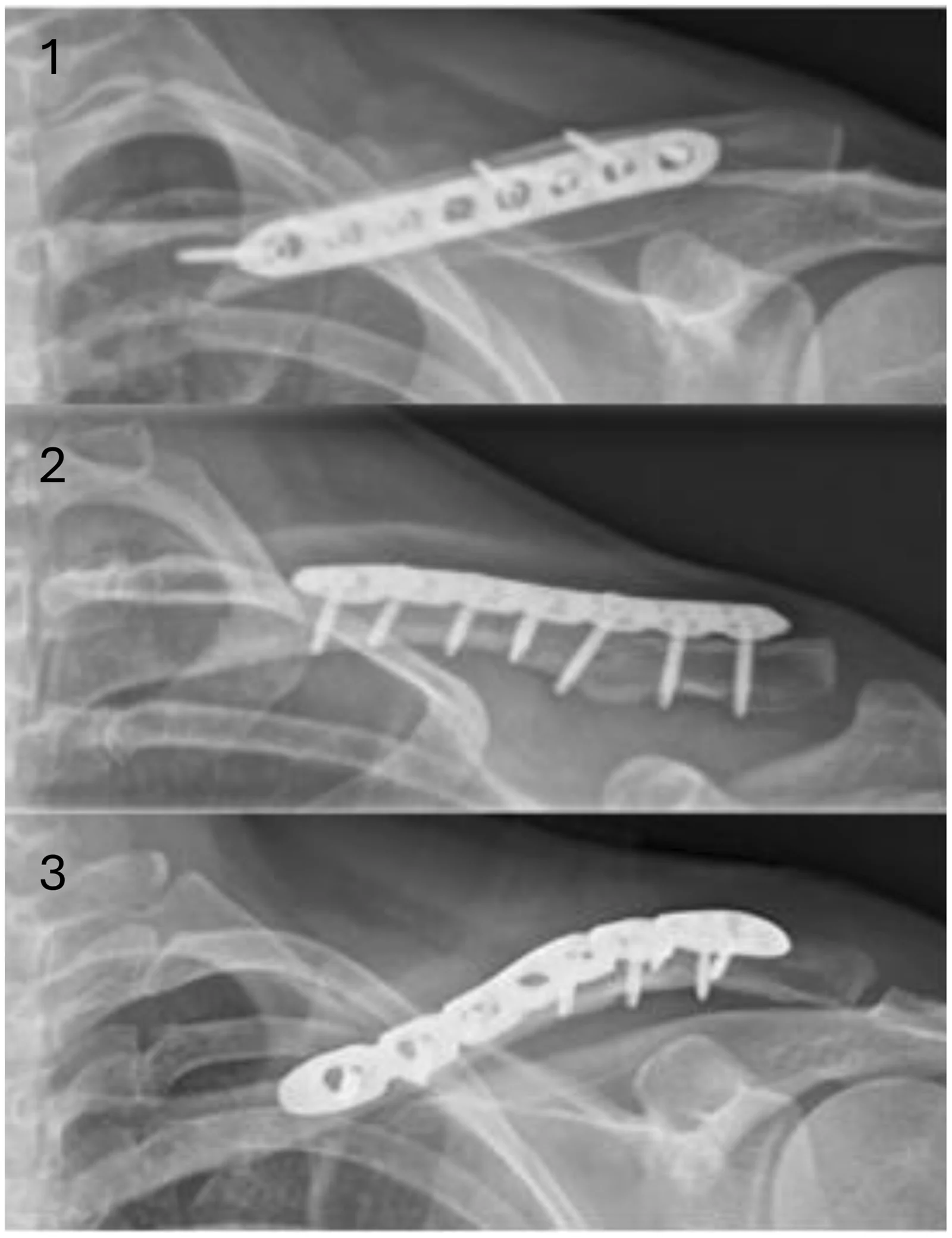
Illustration of the different plate positions: 1 anterior, 2 superior, 3 anterior-superior

Plate fixation can be applied in superior, anterior-superior and anterior techniques (Fig. 1: Illustration of the different plate positions). Advantages and disadvantages of anterior and superior plate placement have already been investigated in previous studies [11–14]. While some studies report no significant difference in functionality [13, 14, others suggest that anterior plate placement may result in superior functional outcomes compared to superior plate placement [11, 12]. Shorter operating times, less intraoperative blood loss, and faster healing rates are described with anterior plate fixation [14]. In contrast, superior plate fixation is associated with more frequent irritation from osteosynthesis material and a higher rate of implant removal [11, 13]. Overall, no significant differences have been observed between the anterior and superior fixation in terms of complication rates or non-union [11, 13, 14]. The anterior-superior plate is an anatomically contoured implant positioned laterally on the superior surface and medially on the anterior surface of the clavicle. Advantages are straightforward implantation, biomechanical stability and reduced plate prominence [15–17]. But studies exploring anterior-superior plate fixation remain scarce.

The main aim of the study is to compare anterior, anterior-superior and superior plate placement in clavicle shaft fractures regarding callus formation as a radiological indicator of bone healing, complication rate and the need for implant removal. We hypothesize that anterior plate fixation results in fewer complications and less frequently implant removal compared to anterior-superior and superior plate fixation. The secondary objective of this study is to compare the frequency of use and the corresponding patient and perioperative characteristics across the different plate positions.

## Materials and methods

This retrospective cohort study was conducted in accordance with the STROBE guidelines (STROBE: Strengthening the Reporting of Observational Studies in Epidemiology) [18]. The study was approved by the Ethical Committee of the Canton Zürich (Study-Nr 2024 − 01809). Data were collected from patients with clavicle shaft fractures who underwent surgical treatment between January 2016 and December 2024 in a Swiss level 1 trauma center. All participants provided written consent for the use of their retrospective data for scientific research in form of a generalized informed consent for retrospective analysis of clinical data. Follow-up was conducted through regular outpatient consultations.

Patients with surgically treated clavicle fractures were identified through an automatic search in the clinical information system. Three authors screened patients for eligibility based on predefined inclusion and exclusion criteria. Inclusion criteria consisted of: (1) clavicle shaft fractures, with or without concomitant injuries, that were treated with plate osteosynthesis; (2) age ≥ 16 years; (3) availability of clinical follow-up data including X-ray; and (4) signed informed consent. Frequent associated injuries comprised traumatic brain injury, thoracic trauma, spinal injury, and extremity fractures, none of which influenced the selection of plate position. Exclusion criteria included patients with non-surgical treatment, fractures of the clavicle not involving the shaft, incomplete data sets, and those who declined to provide informed consent.

Medical records, including operative reports, follow-up documentation, conventional radiographs, CT scans, and intraoperative imaging, were manually reviewed. Anterior plate fixation was predominantly performed using manually contoured locking compression plates (LCPs), whereas anterior-superior and most superior plate fixations were carried out using anatomically precontoured LCPs. Regarding complications, the following were distinguished: pseudarthrosis (nonunion within six months after injury), peri-implant fracture, breakage of screws, screw loosening, secondary dislocation, persistent severe local pain with restrictions in everyday life, infection (early infection within six weeks, late infection after six weeks postoperatively), impaired wound healing and misplacement of osteosynthesis material with the need for further surgery. To assess osseous healing, we examined X-ray images for callus formation and subdivided the occurrence of callus into early (≤ 6 weeks), intermediate (> 6 to ≤ 26 weeks), and late (> 26 weeks).

### Statistics

All statistical analyses were conducted using Python™. Continuous variables were tested for normal distribution (Shapiro–Wilk test) and are presented as mean ± standard deviation or median (interquartile range) as appropriate. Comparisons of means between groups were performed using one-way ANOVA, while medians were compared using the Kruskal–Wallis test. Categorical variables were analyzed with cross-tabulations and tested for group differences using the Chi-square test, or, when expected cell counts were < 5, the Fisher’s exact test. For continuous, non-normally distributed data, the Mann–Whitney-U test was applied. A *p*-value < 0.05 was considered statistically significant.

## Results

A total of 385 cases were identified. 30 patients were excluded due to lack of follow-up examinations. Of the remaining 355 patients, 81.7% (*n* = 290) were male and 18.3% (*n* = 65) were female. Patients ranged in age from 17 to 90 years, with a median of 41 years. Regarding fracture morphology, 30.7% of the patients (*n* = 109) had fractures with two fragments, 30.7% (*n* = 109) had fractures with three fragments, and 38.6% (*n* = 137) had fractures with more than three fragments. The fracture was located on the right side in 48.2% of cases (*n* = 171) and on the left side in 51.8% of cases (*n* = 184).

### Plate position

The majority of patients underwent anterior plate fixation (92.4%, *n* = 328), followed by superior plate fixation (5.6%, *n* = 20) and anterior-superior plate fixation (2%, *n* = 7). The average operating time was 87.9 (± 24.9) minutes for anterior plate position, 105.3 (± 39.3) minutes for anterior-superior plate position, and 92.8 (± 24) minutes for superior plate position (Table 1). There were no statistically significant differences between the three plate positions regarding age (*F* (2, 354) = 0.065, *p* = 0.937), sex distribution (*χ*^2^ (2, *N* = 355) = 5.38, *p* = 0.068), number of fragments (*χ*^2^ (4, *N* = 355) = 3.850, *p* = 0.430) and operation time (*F* (2, 354) = 1.94, *p* = 0.145).

**Table 1:** Frequency of use, age, sex distribution and operation time for the different plate positions (anterior, anterior-superior, superior)

Plate position	Total cases		Operation time		Age (years)		Sex (% Male/% Female)
	(*n*)	(%)	(mean ± SD)	(median)	(mean ± SD)	(median)	
anterior	328	92.4	87.9 ± 24.9	85	43.2 ± 15.7	40	82.3/17.7
anterior-superior	7	2	105.3 ± 39.3	105	44.7 ± 14.3	45	100/0
superior	20	5.6	92.8 ± 24.0	93.5	44.2 ± 18.5	44	65/35

### Complications

The overall complication rate was 6.2% (*n* = 22). Regarding plate position, the complication rate was 5.5% (*n* = 18) for anterior plate placement, 10.0% (*n* = 2) for superior plate placement and 28.6% (*n* = 2) for anterior-superior plate placement. There was a statistically significant difference between the complication rates of the three plate positions (*χ*^*2*^ (2, *N* = 355) = 6.830, *p* = 0.033). When considering only superior and anterior plate placement, no statistically significant difference was found regarding the incidence of complications (*p* = 0.322).

The most frequent complications associated with anterior plate fixation were persistent severe local pain (1.2%, *n* = 4) and infection (0.9%, *n* = 3). In our study, all infections occurred early, within six weeks after surgery. In the anterior-superior plate fixation group, only pseudarthrosis (14.4%, *n* = 1) and peri-implant fracture (14.3%, *n* = 1) were observed. For superior plate fixation, the two recorded complications were screw loosening (5%, *n* = 1) and secondary dislocation (5%, *n* = 1) (Table 2).

**Table 2:** Overview of complications stratified by type and plate position

	anterior		anterior-superior		superior	
	*n*	%	*n*	%	*n*	%
Total complications	18	5.5	2	28.6	2	10
Persistent severe pain	4	1.2	0	0	0	0
Infection	3	0.9	0	0	0	0
Infection + breakage of screws	1	0.3	0	0	0	0
Breakage of screws	1	0.3	0	0	0	0
Screw loosening	0	0	0	0	1	5
Peri-implant fracture	2	0.6	1	14.3	0	0
Secondary dislocation	2	0.6	0	0	1	5
Misplacement of osteosynthesis material	1	0.3	0	0	0	0
Impaired wound healing	2	0.6	0	0	0	00
Pseudarthrosis	2	0.6	1	14.3	0	0

### Callus

Callus was detected in 281 patients (79.2%) during follow-ups with a median after 12.0 weeks (minimum 6 weeks, maximum 52 weeks). The appearance of callus with subdivision into no, early, intermediate and late callus for the three different plate positions is summarized in Table 3. Callus formation did not differ statistically significantly between the three plate positions, neither in the distinction between visible callus formation and no callus (*χ*^2^ (2, *N* = 355) = 2.060, *p* = 0.357) nor in the distinction between the subgroups of callus formation (*χ*^2^ (6, *N* = 355) = 4.970, *p* = 0.597).

**Table 3:** Timing of callus formation according to plate position

Plate position	Total cases (*n*)	Early callus		Intermediate callus		Late callus		No callus	
		(*n*)	(%)	(*n*)	(%)	(*n*)	(%)	(*n*)	(%)
anterior	328	121	36.9	134	40.9	4	1.2	69	21.0
anterior-superior	7	4	57.1	3	42.9	0	0	0	0
superior	20	7	35.0	7	35.0	1	5.0	5	25.0

### Implant removal

A total of 134 out of 355 patients (37.7%) underwent implant removal. When differentiating by plate fixation position, implant removal occurred in 37.2% of patients (*n* = 122) with anterior plate placement, in 57.1% of patients (*n* = 4) with anterior-superior plate placement and in 40.0% of patients (*n* = 8) with superior plate placement. The implant removal was performed after a median of 22.0 months after the initial surgery (earliest after 3 months, latest after 149 months). The duration of implant removal was a median of 41.5 min (minimum 19 min, maximum 277 min) (Table 4). There is no statistically significant difference between the three plate positions regarding the frequency of implant removal, time to implant removal and the duration of the operation (*p* > 0.05). Apart from the 22 cases with the complications described above, the remaining implant removals were carried out electively at the patients’ request due to bothersome osteosynthesis material.

**Table 4:** Frequency, timing and duration of implant removal depending on the plate position

Plate position	Total cases (*n*)	Implant removal (*n*)	Implant removal (%)	Time until implant removal (months, MW)	Duration of implant removal (minutes, MW)
anterior	328	122	37.2	23.4 (22)	48.9 (41)
anterior-superior	7	4	57.1	21.5 (24)	66.2 (42)
superior	20	8	40.0	20.8 (20)	45.6 (46.5)

### Complications after implant removal

Overall, refracture occurred in eleven cases (8.2%) after implant removal. Regarding plate placement, refractures were found in ten patients (8.2%) with an anterior plate position and in one patient (12.5%) with a superior plate position. No refracture was reported by patients with anterior-superior plate position. The incidence of refracture did not differ statistically significantly between the three plate positions (*χ*^2^ (2, *N* = 134) = 0.550, *p* = 0.758).

## Discussion

The present study analyzed 355 patients with surgically treated middle-third clavicle shaft fractures (Allman type I). Most patients received anterior plate osteosynthesis, followed by superior and anterior-superior plate placement. We found no significant differences between the three groups regarding age and sex distribution, number of fracture fragments, or operation duration. This contrasts with a meta-analysis by Ai et al., which reported shorter surgery times for anterior compared with superior plate fixation [14]. Shorter operation times in anterior plating have been attributed to the larger anterior clavicular diameter, facilitating plate adaptation, as well as to differences in plate contouring requirements [14]. In our cohort, comparable operative durations appear plausible, as all approaches provide straightforward exposure and fracture reduction, and increased contouring demands of reconstruction plates used in anterior-superior and superior fixation may offset potential positional advantages. Accordingly, operative duration may be influenced more by surgeons’ experience and implant morphology than by plate position alone.

Moreover, plate placement had no significant influence on callus formation, as a radiological indicator of bone healing, which is consistent with the literature [11]. However, a shorter healing rate with anterior plate placement has also been reported in some studies [14]. Callus formation depends on interfragmentary movements and is not necessarily a reliable marker for healing and biomechanical stability [19]. Precise anatomic reduction with stable osteosynthesis may result in minimal or absent visible callus formation, yet represents the biomechanically most stable construct [19]. Also, we found no difference in the incidence of refracture after removal of an anterior or superior plate. Even though in some cases no callus was visible (e.g. in over 20% of cases with anterior plate placement), good results were seen after implant removal, confirming that callus is not a decisive factor for stability.

Implant removal was independent of plate position in our study. The literature presents different results: some studies indicate increased material removal with superior plate placement [11, 13, while others find no correlation between implant removal and plate position [20]. This suggests that common reasons for implant removal (plate prominence, hardware irritation, a foreign body sensation and cosmetic reasons with a visible plate) [21 may not primarily depend on plate position. The timing and duration of implant removal also did not differ between the three plate placements.

The overall complication rate was 6%, with the most frequently used anterior plate position having the lowest complication rate. A trend towards a higher complication rate was observed in patients with anterior-superior plate placement; however, this finding is of limited significance due to the small number of cases. To date, no studies have been published on this specific comparison in the literature. The higher complication rate could be explained by the complex anatomy of the clavicle bone. The clavicle is a double-curved S-shaped bone and differs between individuals [22, 23]. Bones can be strained and fracture in various ways, including compression, bending, and torsion [22]. Accordingly, fracture reduction of the clavicle is complicated and must be achieved in several planes. Complex fractures and the variable anatomy of the clavicle often make it challenging for the surgeon to make a plate fit adequately [22, 23]. With anterior-superior plate fixation, adjustment of the plate is required in two planes and is therefore potentially more prone to malposition. Comparing only anterior and superior plate placement, no difference was found regarding the occurrence of complications. Several studies came to the same conclusion [13, 14]. In our study, only screw loosening and secondary dislocation occurred with superior plate placement, whereas persistent severe pain, impaired wound healing, infection within six weeks postoperatively, misplacement of osteosynthesis material, peri-implant fractures, breakage of screws and pseudarthrosis were also described in anterior plate placement. Although more diverse complications occurred with anterior plate placement, the complication rate was still the lowest.

## Limitations

There are some limitations in our study that should be considered when interpreting the results. First, the majority of cases were treated with anterior plate fixation (92.4%, *n* = 328) compared to superior plate fixation (5.6%, *n* = 20) and anterior-superior plate fixation (2%, *n* = 7). Part of the results are of limited significance due to the small number of cases involving anterior-superior plate placement. The absolute number and type of complications were higher with anterior plate placement due to the differently distributed plate positions. Also, the study assumes that surgical techniques are similar across different positions, but technical differences not directly recorded in the protocol (e.g. incision size and dissection techniques) could influence the surgical outcome. A more detailed analysis of the surgical technique could be helpful in this regard. Callus formation, indicative of secondary bone healing, was assessed; however, fractures may also heal via primary bone healing without visible callus. Furthermore, the assessment of callus was based on conventional x-rays. The quality of the x-ray images depends on the radiology assistant, and the radiological interpretation of callus depends on the examiner. There are standardized follow-up appointments (6 weeks, 3 months, 6 months, 12 months postoperatively), but these have sometimes varied slightly for various reasons and can influence the timing of callus appearance. Finally, patient reported outcome scores, and clinical outcomes were not analyzed. We feel that the thorough clinical examination and documentation of complications contributed to the reliability of our main conclusions.

## Conclusion

Anterior, anterior-superior, and superior plate fixation represent established surgical treatment options for clavicula shaft fractures. No significant differences were observed between plate positions with regard to patient demographics, duration of surgery, or timing of callus formation. Although anterior and superior plate positioning were associated with low complication rates in our dataset, these findings should be interpreted with caution given the absence of significant group differences and the imbalance between treatment groups. Implant removal remained necessary in a proportion of cases across all techniques. Further prospective studies, especially focusing on anterior-superior plate fixation and incorporating patient-reported outcome measures and clinical endpoints, are warranted to better define long-term surgical outcomes.

## Data Availability

No datasets were generated or analysed during the current study.

## References

[1] Postacchini F, Gumina S, De Santis P, Albo F. Epidemiology of clavicle fractures. J Shoulder Elb Surg. 2002;11(5):452–6. 10.1067/mse.2002.126613. 12378163

[2] Nordqvist A, Petersson C. The incidence of fractures of the clavicle. Clin Orthop Relat Res. 1994;(300):127–32. 8131324

[3] Allman FL. Fractures and ligamentous injuries of the clavicle and its articulation. J Bone Joint Surg Am. 1967;49(4):774–84. 6026010

[4] Axelrod DE, Ekhtiari S, Bozzo A, Bhandari M, Johal H. What is the best evidence for management of displaced midshaft clavicle fractures? A systematic review and network meta-analysis of 22 randomized controlled trials. Clin Orthop Relat Res. 2020;478(2):392–402. 10.1097/CORR.0000000000000986. PMC7438117 31574019

[5] Amer K, Smith B, Thomson JE, et al. Operative versus nonoperative outcomes of middle-third clavicle fractures: a systematic review and meta-analysis. J Orthop Trauma. 2020;34(1):e6–13. 10.1097/BOT.0000000000001602. 31851115

[6] Fayed MA, Mohammed AT, El-Gritly MET, El Deriny A. Plate fixation versus conservative treatment for displaced midshaft clavicle in adults. Med Updates. 2025;0(0):0–0. 10.21608/muj.2025.410729.1245.

[7] von Rüden C, Rehme-Röhrl J, Augat P, et al. Evidence on treatment of clavicle fractures. Injury. 2023;54:110818. 10.1016/j.injury.2023.05.049. 37217399

[8] Vannabouathong C, Chiu J, Patel R, et al. An evaluation of treatment options for medial, midshaft, and distal clavicle fractures: a systematic review and meta-analysis. JSES Int. 2020;4(2):256–71. 10.1016/j.jseint.2020.01.010. PMC7256900 32490412

[9] Houwert RM, Wijdicks FJ, Steins Bisschop C, Verleisdonk EJ, Kruyt M. Plate fixation versus intramedullary fixation for displaced mid-shaft clavicle fractures: a systematic review. Int Orthop. 2012;36(3):579–85. 10.1007/s00264-011-1422-4. PMC3291769 22146919

[10] Smith SD, Wijdicks CA, Jansson KS, et al. Stability of mid-shaft clavicle fractures after plate fixation versus intramedullary repair and after hardware removal. Knee Surg Sports Traumatol Arthrosc. 2014;22(2):448–55. 10.1007/s00167-013-2411-5. 23370985

[11] Formaini N, Taylor BC, Backes J, Bramwell TJ. Superior versus anteroinferior plating of clavicle fractures. Orthopedics. 2013;36(7):e898–904. 10.3928/01477447-20130624-20. 23823047

[12] Rivera-Saldívar G, Manrique-Ávila JA. Superior versus anteroinferior plating for mid-shaft clavicle fractures: a randomized clinical trial. Cir Cir. 2024;92(2):141–9. 10.24875/CIRU.22000562. 38782391

[13] Nourian A, Dhaliwal S, Vangala S, Vezeridis PS. Midshaft fractures of the clavicle: a meta-analysis comparing surgical fixation using anteroinferior plating versus superior plating. J Orthop Trauma. 2017;31(9):461–7. 10.1097/BOT.0000000000000936. 28708779

[14] Ai J, Kan SL, Li HL, et al. Anterior inferior plating versus superior plating for clavicle fracture: a meta-analysis. BMC Musculoskelet Disord. 2017;18(1):159. 10.1186/s12891-017-1517-1. PMC5395806 28420364

[15] VanBeek C, Boselli KJ, Cadet ER, Ahmad CS, Levine WN. Precontoured plating of clavicle fractures: decreased hardware-related complications? Clin Orthop Relat Res. 2011;469(12):3337–43. 10.1007/s11999-011-1868-0. PMC3210289 21416203

[16] LCP Superior Anterior Clavicle 3.5. https://www.aofoundation.org/approved/approvedsolutionsfolder/2009/lcp-superior-anterior-clavicle-35. Accessed 4 Feb 2026.

[17] Eden L, Doht S, Frey SP, et al. Biomechanical comparison of the locking compression superior anterior clavicle plate with seven and ten hole reconstruction plates in midshaft clavicle fracture stabilisation. Int Orthop. 2012;36(12):2537–43. 10.1007/s00264-012-1671-x. PMC3508054 23073924

[18] von Elm E, Altman DG, Egger M, et al. The Strengthening the Reporting of Observational Studies in Epidemiology (STROBE) statement: guidelines for reporting observational studies. J Clin Epidemiol. 2008;61(4):344–9. 10.1016/j.jclinepi.2007.11.008. 18313558

[19] Claes LE, Heigele CA. Magnitudes of local stress and strain along bony surfaces predict the course and type of fracture healing. J Biomech. 1999;32(3):255–66. 10.1016/s0021-9290(98)00153-5. 10093025

[20] Mullis BH, Jeray KJ, Broderick S, et al. Midshaft clavicle fractures: is anterior plating an acceptable alternative to superior plating? Eur J Orthop Surg Traumatol. 2023;33(8):3373–7. 10.1007/s00590-023-03563-5. 37130985

[21] Davis BP, Shybut TB, Coleman MM, Shah AA. Risk factors for hardware removal following operative treatment of middle- and distal-third clavicular fractures. J Shoulder Elb Surg. 2021;30(3):e103–13. 10.1016/j.jse.2020.06.034. 32663568

[22] Zheng Y, Li J, Tam AYC, et al. Finite element modeling of clavicle fracture fixations: a systematic scoping review. Med Biol Eng Comput. 2025;63(6):1585–607. 10.1007/s11517-025-03294-1. PMC12106152 39873960

[23] Vancleef S, Herteleer M, Carette Y, et al. Why off-the-shelf clavicle plates rarely fit: anatomic analysis of the clavicle through statistical shape modeling. J Shoulder Elb Surg. 2019;28(4):631–8. 10.1016/j.jse.2018.09.018. 30609957

